# The Treatment of Empyema of the Antrum of Highmore

**Published:** 1895-04

**Authors:** Robert Levy

**Affiliations:** Denver, Colo.


### THE TREATMENT OF EMPYEMA OF THE ANTRUM OF HIGHMORE.

ROBERT LEVY, M. D., DENVER, COLO.

My excuse for offering anything upon the treatment of the empyema of the antrum of Highmore, at a time when this subject is receiving almost as much attention in medical literature as appendicitis and curetting of the uterus, is the same which, I take it, urges others to write on the subject. This reason is undoubtedly in the fact that much dispute exists among various practitioners as to the ease of accomplishing a cure of an affection which has seemed to me one of the most difficult to remedy. Dentists never fail in their treatment of empyema of the antrum (if we believe all they say and write), and I believe they are honest when they make such a statement. I believe they are honest because they come in contact with a different class of cases than do rhinologists. Their cases may, in the great majority of instances, be attributed to a diseased tooth, which, when removed, takes away the source of irritation, and the antrum rapidly becomes well, under very little treatment. The rhinologist, however, comes in contact with a class of cases which, having perhaps been latent for a long period of time, may be due—and usually is—to one of a great variety of causes other than disease of a tooth. Our failure in the treatment of these cases I believe to be due, primarily, to two causes—one, that we do not always recognize the important cause; the other, that we fail to remember the exact anatomical conditions that obtain here. Especially do we fail to remember that the only outlet for accumulations in the antrum is an opening in its upper portion, as well as to appreciate that the lowest portion of the antrum—that portion from which drainage is best obtained—is considerably below the floor of the nasal cavities.* The causes which frequently escape our observation and which must first be recognized and removed before a cure can be affected are nasal complications, such as hypertrophied middle turbinate and nasal polypi, the presence within the antral cavity of a variety of tumors or of bony laminæ, or of for-

*Zuckerandl. II. Band. 1802.
Fraenkel. Gefrierdurchschnitte. 1891.

eign bodies, or polypoid mucous membrane. Another cause which, although not very common, may exist and become a source of a continuous empyema of the antrum is a similar disease of the frontal sinus or anterior ethmoidal cells. I am convinced that, owing to the close relation between the openings of the frontal sinus and anterior ethmoidal cells and the opening of the antrum of Highmore in the hiatus semilunaris, a discharge may readily pass from the upper cavaties into the antrum, and keep up, even though most thorough antral treatment be instituted, a chronic empyema here. The causes above mentioned include only those which are apt to be overlooked, and which, having been overlooked, offer one explanation for the failure to cure. As for the recognition of the anatomical peculiarities of the part, failure to cure depends not infrequently upon the fact that an opening has been made in another than the lowest portion of the antrum, and, in connection with this, that drainage is badly affected. Having satisfied ourselves as to the cause of the disease, we must proceed to its removal.

Together with the proper treatment of associated disease, whether intranasal or constitutional, or of other cavities, the antrum must be opened for the purpose of exploration, the removal of a tumor or of other morbid condition within the cavity, proper drainage and proper irrigation. The operation which I believe to be the best is the one similar to that described by Moreau Brown,* and which I performed previous to seeing his paper. It consists in drilling, by means of an electric burr, into the antrum, at a point between the roots of the second bicuspid and first molar, just at the alveolar apophysis. An opening here I believe to be a better operation than one through the canine fossa, for it can be enlarged to any extent, and possesses an advantage in being in the lowest portion of the antrum. The opening through the alveolar process, after the removal of the tooth, furnishes a good operation, so far as drainage is concerned, but is less efficient for any radical treatment or thorough exploration which may become necessary. Having opened the antrum in the situation indicated, a probe or electric search light, or even the finger, may be introduced. Should any intra-antral disease exist, such as a tumor, presence of foreign body, or change in the mucous membrane, it

*Journal of L. R. & O., May, 1893, will be readily detected and can as readily be removed. In the majority of instances of long standing antral disease, it has been my experience to find polypoid degeneration of the lining mucous membrane. It is my practice to curette this membrane thoroughly by means of a modified ear curette. After operating, it is well to irrigate thoroughly with a solution of bichloride of mercury, one to 4,000, and then to introduce into the opening a drainage tube. I have had constructed a tube of hard rubber, with a flange so arranged as to prevent it from passing into the cavity. The pressure of the cheek prevents its falling out. I desire to mention two points in the construction of the tube. The first concerning the flange. This is made so as to fit closely to the mucous membrane surrounding the opening without affording an unequal pressure. The second is in regard to the length of the tube, which I believe should reach a short distance within the antrum. If the tube is too short, the mucous membrane of the antrum rapidly covers over its extremity. A small opening may be made a short distance from its extremity, for better drainage. The future treatment depends more, I believe, upon the method of irrigating than upon the remedies used. A syringe forcing its contents into the antrum fails to cover the entire antrum with the solution, and a method of irrigation should be used which will allow the solution to enter slowly, not forcing it out, but allowing it to overflow through the natural opening in the nose. The best means for accomplishing this is the fountain douche, to which can be attached a fitting for the drainage tube. The remedies used may be divided into three classes—first, cleansing and antiseptic; second, stimulating; third, protective. Among the firstclass may be mentioned solution of bichloride of mercury, 1 to 4,000; hot saturated solution of boracic acid: peroxide of hydrogen, or pyrozone in the strength of 1 part to 8, with gradual increase; listerine, alone or with pyrozone; carbolic acid, three per cent. Among the second class I simply mention solutions of iodine, nitrate of silver, sulpho-carbolate of zinc, chloride of zinc. For permanent antiseptic and protective applications may be used powdered iodol, aristol or boric acid, salol, which may be injected in a liquid form by applying a mild heat to it, and aristol suspended in sterilized oil.

DISCUSSION.

DR. BLACK—I notice that the Doctor makes his opening as low down as possible. The first operation I performed for diseases of the antrum, I made the opening in the canine fossa, and I was very much annoyed by the mucous membrane covering over the end of my tube. This led me to believe that an operation lower down would be better. In making the operation as low down as possible, it has a two-fold purpose—the tube is more accessible and remains in position better. I have a tube which I have arranged, which, I think, is very convenient—a simple round gold tube of any size and diameter that you desire. With a pair of scissors, make some snips, about $\frac{1}{16}$ inch deep and $\frac{1}{16}$ inch apart, in the end of it; bend these out to form a flange; take a piece of soft rubber tubing $\frac{1}{2}$ inch long; snip a hole in the side, and force tube through it up to flange. The sides of rubber tube project over end of gold tube, protecting it from entrance of food and affording a soft cushion to the mucous membrane. By slitting the rubber tube, you have a kind of valve protection which prevents the introduction of food, but which will allow a very easy introduction of the point of a syringe. I do not believe that I have anything to add. This subject is a very interesting one to me, and has been very generally overlooked. There is no doubt that a number of patients have been treated for other diseases without this trouble being recognized.

DR. WHITNEY—I would like to ask how long it will take to cure the disease?

DR. LEVY—In regard to the appliance of Dr. Black, I think it is a very excellent suggestion, but at the same time, I believe that the matter of food entering the antrum is very largely exaggerated. Even when the opening is made through the alveolar process, the danger from food entering is not very great. Formerly, we had all sorts of appliances to prevent its entrance, but I have never been troubled in any way in this particular.

The length of time consumed in the treatment of a case is variable. I am glad the Doctor mentioned that, from the fact that I have lost a great many patients before they got well. The fact is, the best authorities that I can find say that two months is the very shortest time in which a chronic case of empyema of the antrum can be cured. They never get well under taht, and sometimes not under a year or eighteen months.

